# Effect of built environment on BMI of older adults in regions of different socio-economic statuses

**DOI:** 10.3389/fpubh.2023.1207975

**Published:** 2023-07-07

**Authors:** Peng Zang, Kaihan Chen, Haifan Zhang, Hualong Qiu, Yun Yu, Jianwen Huang

**Affiliations:** Department of Architecture, Guangdong University of Technology, Guangzhou, China

**Keywords:** body mass index, built environment, linear regression, older adults, obesity, socio-economic status

## Abstract

**Background:**

Numerous studies have ignored the influence of underdeveloped urban surroundings on the physical health of China’s ageing population. Lanzhou is a typical representative of a less developed city in China.

**Methods:**

This study investigated the relationship between body mass index (BMI) and built environment amongst older adults in regions of different socio-economic statuses (SES) using data from medical examinations of older adults in Lanzhou, as well as calculating community built environment indicators for regions of different SES based on multiple linear regression models.

**Results:**

Results showed that age and underlying disease were negatively associated with overall older adult BMI in the study buffer zone. Land use mix, number of parks and streetscape greenery were positively associated with older adult BMI. Street design and distance to bus stops were negatively connected in low SES regions, but population density and street design were negatively correlated in high SES areas.

**Conclusion:**

These findings indicate that the built environment of SES regions has varying impacts on the BMI of older persons and that planners may establish strategies to lower the incidence of obesity amongst older adults in different SES locations.

## 1. Introduction

According to the seventh census of the China Bureau of Statistics, the population aged 60 and over in China is 264.02 million, or 18.70% of the total population. With 190.64 million people aged 65 and older, or 13.50% of the population, China will enter a profoundly ageing society (1). Building an appropriate community constructed environment for the older adult is an important need for an ageing society to improve the living environment of the older adult and increase their quality of life. The greatest challenge facing the older adult as the body’s functions begin to decline with age is health, especially chronic diseases such as cardiovascular disease, type II diabetes, hypertension and colon cancer (2). Obesity is an important cause of these chronic diseases (3, 4), and it seriously impacts health and quality of life of older adult individuals. Body mass index (BMI) is a commonly used international measure of body fatness and health, and a higher BMI indicates a higher likelihood of being overweight. The BMI of older adults is influenced by some factors, such as built environment (5).

The built environment consists of several components, including land use, transport planning and urban design (6). The built environment has been categorized into five dimensions: density, diversity, design, destination accessibility and distance to transit; they form the 5D model of the built environment (7). The 7D model was developed by introducing demand management and demographics to quantify the built environment (8). The development of technology in recent years has enabled scholars to add a more comprehensive description of the built environment by introducing normalized difference vegetation index (NDVI) (9, 10) and street-level greenery (11–13). Many studies have shown a substantial correlation between the built environment and obesity (14, 15). The design of built environments may reduce the incidence of overweight and obesity amongst people by offering walkable designs and supporting physical activity and a healthy eating environment (16). Existing studies have focused on the relationship between built environment and BMI by segmenting groups of older adults of different socio-economic statuses (SES) (17, 18). The built environment in high SES areas has less impact on older adult’s BMI than in low SES areas, and higher accessibility to public sporting facilities and better public sporting services and road connectivity near where older adults live increase the probability of transport-based physical activity and thus decrease the risk of obesity (19). The density of road intersections, the number of facilities in parks near the place of residence, the number of work and residential exercise facilities and the availability of recreational exercise areas are significantly and negatively associated with BMI levels (20). The built environment of the community has a significant effect on BMI (21).

The presence of public green spaces may provide physical exercise for older adults (22, 23) and can positively impact psychological well-being of older adults (24), all of which may lead to changes in BMI (25, 26). Recent studies have shown that streetscape green ratings are a more objective indicator of the influence of green space on senior citizens (11–13, 27). Therefore, the effect of streetscape greenness on BMI in older adults needs to be explored.

The relationship between the community built environment and health has received attention and research in many developed countries (28). However, most previous research on the relationship between the community built environment and obesity has been conducted in developed western countries. A thorough study of the physical environment factors of adult weight status revealed that Asian nations have undertaken less research on this issue (29). However, these findings are not necessarily applicable to developing countries. Previous studies have mainly focused on more developed cities (30–32), and less in underdeveloped areas, especially industrial cities in underdeveloped areas. In the past, the research on the health of built environment in China’s industrial cities focused on more developed industrial cities, such as Tianjin, Wuhan and other developed cities (33). Lanzhou is an important central city in western China. After the reform and opening up of the People’s Republic of China, Lanzhou is one of the fastest growing cities in China, an industrial city with distinctive characteristics and an immigrant city. The central urban area of Lanzhou is facing prominent environmental contradictions, and the construction of local environmental facilities cannot meet the physical activity needs of urban residents (34). It has certain representativeness in cities with backward economy compared with developed cities. Research on the relationship between community built environment and BMI based on older age groups is also slightly weak in China’s increasing population ageing. As Lanzhou has a large number of immigrants in the process of urban development, the influx of a large number of immigrants in the same period has made the current base of the older adults population in Lanzhou high. The older adults population in Lanzhou accounts for 16.56% of the total population. The characteristics of urban aging are distinct, with a large base, rapid growth, aging and other characteristics. The health problems of the older adults population in Lanzhou are very worthy of attention.

This study aims to explore the relationship between the built environment and the BMI of older adults in Lanzhou City. It intends to address the following three questions: (1) How does the built environment impact older adults’ BMI in Lanzhou? (2) How does the built environment in regions with different socio-economic statuses (SES) affect the body mass index (BMI) of older adults in Lanzhou? (3) How does the street greening rate affect the BMI of older adults in Lanzhou? Through exploring the three questions above, we aim to provide a more objective picture of the impact of the built environment on the BMI of older adults for planners and policymakers to consider when designing for ageing.

## 2. Literature review

### 2.1. Research related to the impact of the built environment on the behavior of older adults

Scholars have demonstrated that the built environment can impact human behavior (35) by attracting people to active travel and thus reduce BMI (36). Providing more physically active places, such as pavement design and streets with higher circulation (37, 38), traffic safety (39), aesthetics (40) and good pedestrian infrastructure (39) can promote healthy behaviors (41) and thus reduce BMI. The proximity and availability of facilities associated with physical activity may be necessary for supporting high-intensity physical activity (42). The built environment may substantially affect the physical and mental health of older adults (43–45). In the current study, density (46), diversity (47), accessibility (48), safety (49), green space (50), and aesthetics (51) have all positively influenced BMI in older adults (14).

Green spaces or parks in cities may lower the BMI of older adults, and prior research has shown that more green public places can encourage older adults to engage in physical activity (52–54). Some studies have demonstrated that streetscape green visibility is an important indicator for describing green spaces (55), and it has only been discovered and applied by scholars in recent years to assess the quality of green spaces (56–58). Street green vision is a method of capturing and identifying street-level images to determine the exposure of greenery in a neighborhood, and it provides an objective view of the green environment from a human perspective (13). Studies have found that higher street greenness is associated with a higher propensity for older adults to exercise (12), which in turn reduces obesity (59, 60).

The number and proximity of recreational and transport amenities, such as parks and green spaces, might impact the inclination of older adults to travel and engage in physical activity (54, 61). Distance to a park and distance to a bus stop have been shown to be negatively associated with older adults’ activity, and both environmental factors can have a more significant impact on older adults than on younger people (62). In some studies in the Americas, the propensity for older adults to travel was greater when the number of parks was greater. In some studies in Asia, the effect was negative for older adults. A study in Shanghai, China found that an increase in green open spaces such as parks, rivers and squares significantly reduced the BMI of middle-aged and older adults, as did with an increase in the land use mix (2).

### 2.2. Studies related to the impact of the built environment on BMI in older adults

In urban planning and obesity, the link between BMI and built environment in older age groups is a major concern. Studies have shown that higher neighbourhood density, intersections and services are associated with a decreased obesity risk (63, 64). A prior study showed that walking scores were negatively associated with BMI (14). Increasing the land use mix promotes more leisure trips for older adults living in these areas, which reduces BMI in older age groups (65). Similarly, a long-term longitudinal study found that higher densities of land use for retail, churches and recreational and leisure facilities in the neighborhood were more likely to reduce older adults’ BMI (66). Some researchers have found no evidence of an association between neighborhood design and BMI, but most existing studies confirm that street connectivity positively impacts health-related outcomes for residents (67). Cities living in polycentric areas can influence the probability of obesity by affecting movement through multiple pathways (68). Better pedestrian-friendly street design can increase walking and thus reduce BMI in older age groups (69).

Some scholars have demonstrated a facilitative effect of the street environment on the physical activity of older adults in areas with high SES (70, 71). Neighborhoods with low SES lack relevant infrastructure due to pollution, which results in a negative association between the built environment and physical activity in older adults (35). The BMI of older adult groups in low SES regions tends to be higher than that of older adult groups in high SES areas because the latter are better equipped to choose their condition preferences. By contrast, older adults in low SES regions tend to be passively influenced by their environment (72).

The analysis above indicates that research on the influence of the built environment on BMI has concentrated mostly on developed nations and less on developing countries, with the exception of some studies in China that revealed a negative effect of population density on BMI, unlike studies in Europe and America (73, 74). A gap exists in research on the relationship between the built environment and BMI in older Chinese populations, and more research is needed to validate the relationship between BMI and environment in older populations. Existing research in China is insufficient to support the universal application of the theory, and community environment research on low SES populations is few. Therefore, this study aims to explore the influence of the built environment on the BMI of Lanzhou’s older population.

## 3. Materials and methods

### 3.1. Study setting and sample

**Figure 1:**
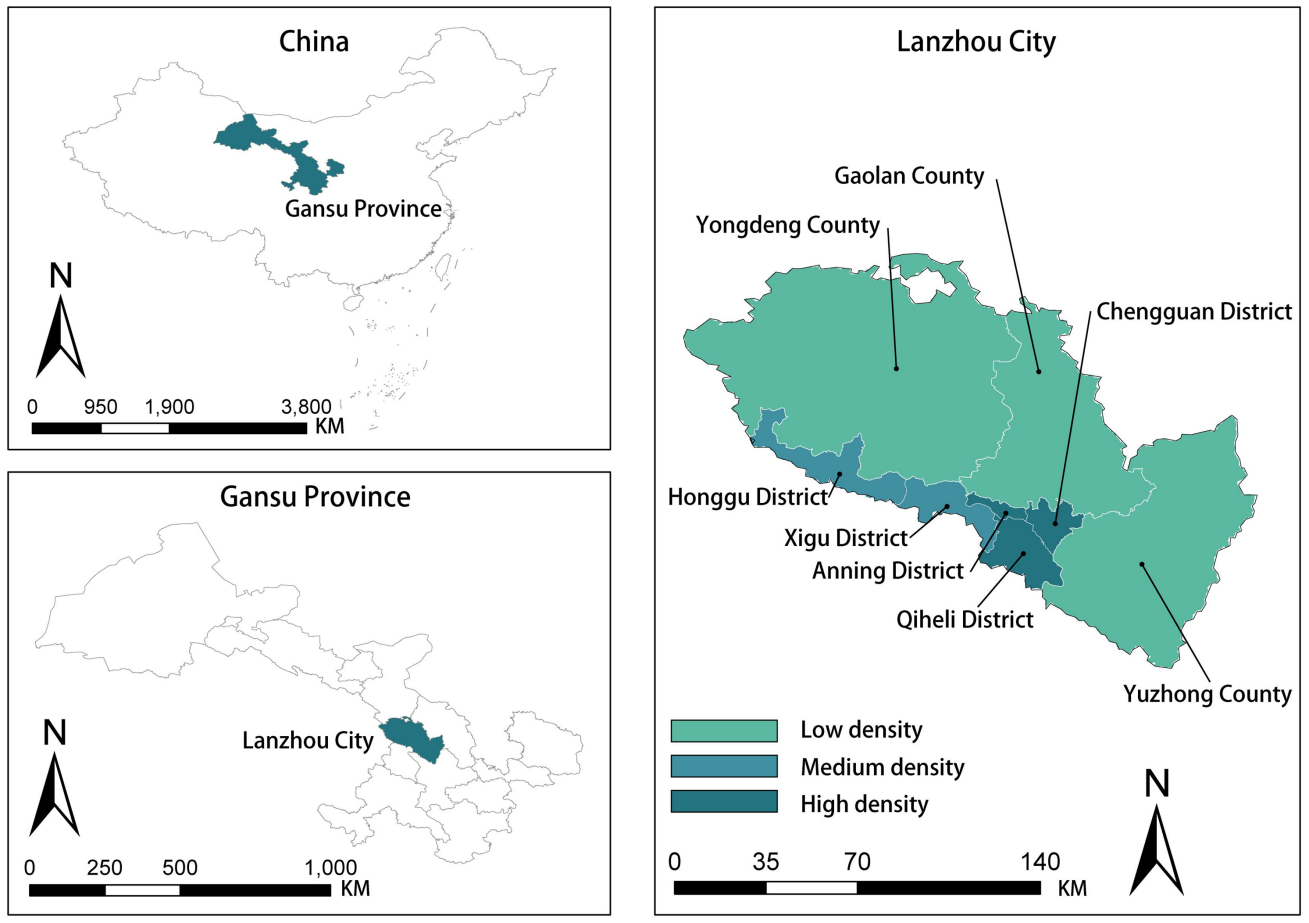
Location of Lanzhou City.

**Figure 2:**
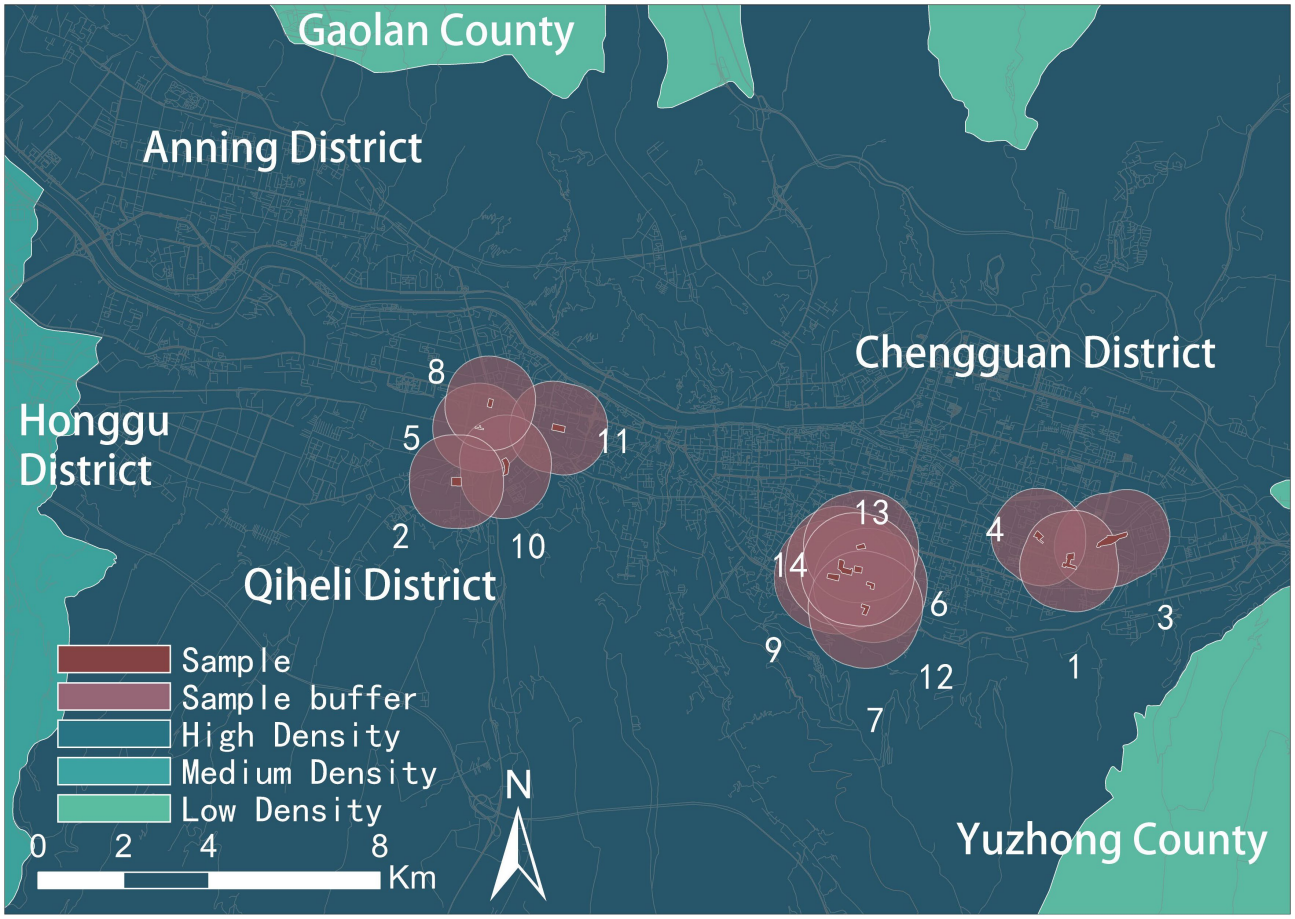
Research sample screening diagram.

**Figure 3:**
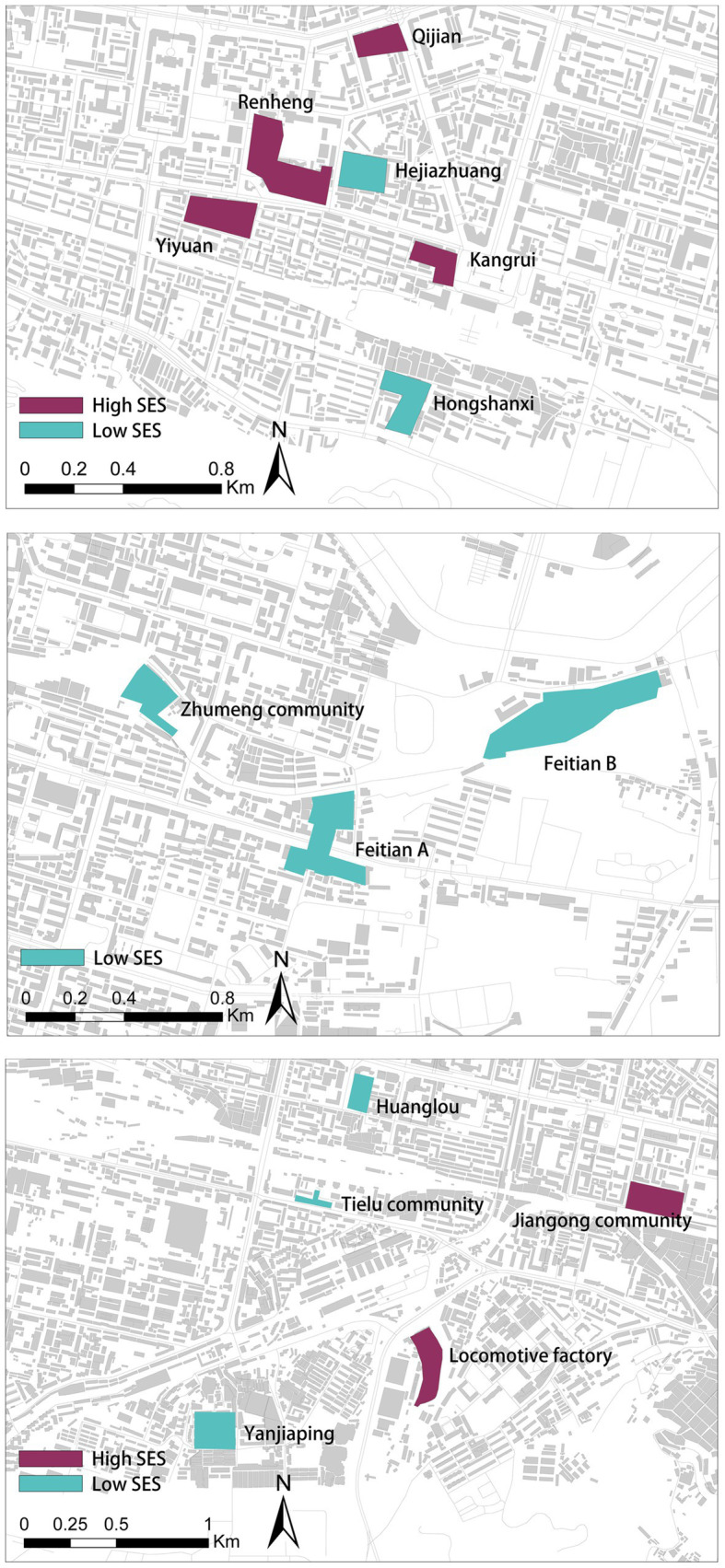
Status of socio-economic indicators for each sample district.

This research is based on the 2021 medical examination reports of middle-aged and older adult Lanzhou Community Hospital residents. The survey data included the respondent’s health condition and personal information, such as his or her residence, height, weight, age and gender. All personal information was anonymized for protection and confidentiality purposes. Data were collected from a total of 1773 older adults. Fourteen communities were selected in Chengguan and Qilihe districts based on density and socio-economic indicators classified by local housing prices (Figures 1, 2). Figure 3 shows the status of socio-economic indicators for each sample area. The built environment data within the buffer zones were calculated using open-source street data, POI data and building information data from Gaode Map.^1^

Lanzhou is the capital city of Gansu Province, located in western China. According to the Lanzhou Municipal Bureau of Statistics, during the seventh national census, the population of people aged 65 and above in Lanzhou City accounted for 11.70% of the total population (75). The number of older adults people aged 65 and above in Lanzhou City reached 510,055 by the end of 2020. It accounts for 11.70% of the resident population, which has exceeded the ‘aging society’ defined by the World Health Organisation. According to the WHO’s definition of ageing society, Lanzhou has entered an ageing society (76).

### 3.2. Measures

BMI: The BMI (kg/m^2^) was determined using self-reported height and weight. In this research, BMI served as the dependent and continuous variable.

Built environment: This research examined 10 environmental factors, including the “5Ds” built environment indicator variables of population density, intersection density, land use mix, distance to parks and distance to bus stations (7). Five variables were also considered: number of parks, street connectivity, number of bus stops, number of pedestrian bridges and streetscape greenery. The built environment factors utilized in this study were selected based on a review of pertinent research (77). Land use mix: The average distribution of land use categories was measured to compute the land use mix or entropy. We identified seven land use categories more relevant to senior travel: residential, commercial, office, healthcare, public services, recreation and education (78). The following calculation was made:


(1) $$Land\;use\;mix=-\sum \frac{i*\left[(p_{i}*\ln\left(p_{i}\right)\right]}{\ln\left(n\right)}$$

where $p_{i}$ is the proportion of specified land use to overall land usage.

Green Vision: The quantity of street vegetation observed by pedestrians at eye level was determined using BSV pictures. A sampling point was created every 50 m based on previous studies to acquire street views (13, 55). The Python Open CV library was used to perform deep learning on the captured streetscape images and extract the greenness. The formula was as follows:


(2) $$\begin{array}{c} Greenviewindex=\sum 4i=1\;Greenerypixelsi/\sum 4i\\ =1\;Totalpixelsi. \end{array}$$

Demographic socio-economic indicators: Individual factors such as age, gender, duration of light physical activity and underlying medical conditions were also considered possible extra variables. Underlying conditions included hypertension, diabetes and coronary heart disease. This information was derived from the survey results.

Research method: The linear regression model was used to discuss the relationship between the built environment of the community and the BMI of the older adults. First, Pearson correlation analysis was performed on all variables. Then stepwise regression is performed on the overall area, high SES area and low SES area, respectively. We established three linear regression models. Model 1 tested the impact of the overall community built environment on the BMI of the older adults. Models 2 and 3 distinguished the impact of the built environment on the BMI of the older adults in communities under different SES. All analyzes were performed using SPSS 26.0 (Statistical Products and Services solution). Then, the multicollinearity test was performed on the data, and if VIF < 5, there was no multicollinearity in the data set, and the significance level was set at *p* < 0.05.

## 4. Analysis

### 4.1. Descriptive statistics

This research includes information from 1773 individuals; we used univariate descriptive statistics to describe the profile of the study sample. Table 1 lists the information for the present study sample. The average age was 71.3 years old, 57.8% were female, the mean BMI was 24.27 kg/m^2^, 52% had underlying medical conditions and the mean weekly duration of light physical exercise was approximately 84 min. In the built environment data, the mean value of population density was 0.07 10^4^ persons/km^2^, the mean value of land use mix was 0.65, the density of road intersections was 26.26 km^2^, the closest bus stop was 124.13 metres away and the distance to the nearest park was 319.24 m.

**Table 1:** Descriptive statistics.

Variable	Description	Mean	SD
Dependent variable			
BMI	Dividing the respondent’s weight (in kilograms) by the square of the height (in meters)	24.275	3.106
Sociodemograpagic variable			
Gender	Gender of respondents; 1 = female, 2 = male	1.42	0.494
Age	Age of interviewees	71.39	7.733
SES	Socio-economic status; 0 = low SES, 1 = high SES	0.56	0.497
Diseases	Basic disease, such as hypertension and diabetes; 0 = no, 1 = yes.	0.52	0.500
Physical activity	Respondents’ reported hours of physical activity per day (in minutes)	84.43	55.031
Built environment variable			
Land use diversity	The proportion of the i-th land use and N is the total number of land use categories. Seven land uses are investigated (*N* = 7): residential, office, commercial, medical, entertainment and public services.	0.653	0.123
Population density	The district’s population density (unit: 10^4^ persons per km^2^)	0.071	0.009
Road intersection density	Density inside a community at a street intersection (unit: 1 km^2^)	26.264	6.454
Street connectivity	Density inside a community at a street intersection (km/km^2^)	1.945	0.392
Bus stop distance	The shortest distance between the test site and the bus stop (meter)	124.139	110.604
Park distance	The park is located at the least distance from the sample plot (meter)	319.240	266.534
Number of parks	The number of groups inside a 1 km buffer zone	1.41	0.939
Number of overpasses	The number of overpasses inside a 1 km buffer zone	3.09	1.741
Number of bus stops	The number of bus stops inside a 1 km buffer zone	33.71	9.467
Streetscape greenery	The greenery covering in the BSV photos represents the status of vegetation from the perspective of a pedestrian	0.163	0.022
Sample size	1773		

### 4.2. Bivariate analysis of the built environment

Based on the empirical research methods, we also used Pearson to explore the relationship between BMI and the built environment. Table 2 displays the Pearson correlation impacts of the built environment on BMI in the older adults, which is broken down into three sections: (1) the effects in the whole research region, (2) the effects in the low SES area and (3) the effects in the high SES area. In order to explore the impact of the built environment on the BMI of the older adults, a total of three models of Pearson correlation were established, namely the entire study area, low SES area and high SES area. Only the variables that have a significant impact on the BMI of the older adults are shown in the table, and the Pearson correlation of all variables will be shown in the Supplementary Appendix.

**Table 2:** Pearson’s bivariate analysis of built environment and BMI in older adults.

	Total area	Low SES area	High SES area
	*R*(value of *p*)	*R*(value of *p*)	*R*(value of *p*)
Sociodemograpagic variable			
Gender	0.032(0.181)	−0.019(0.602)	0.082**(0.01)
Age	−0.169**(0.000)	−0.164**(0.000)	−0.155**(0.000)
Diseases	0.210**(0.000)	0.223**(0.000)	0.203**(0.000)
Physical activity	−0.016(0.502)	−0.04(0.269)	0.005(0.884)
Built environment variable			
Land use diversity	0.003(0.887)	0.055(0.122)	0.012(0.697)
Population density	−0.068**(0.004)	0.002(0.953)	−0.097**(0.002)
Road intersection density	0.098**(0.000)	−0.022(0.531)	0.073*(0.021)
Street connectivity	−0.011(0.644)	−0.072*(0.045)	0.047(0.142)
Bus stop distance	0.100**(0.000)	−0.011(0.757)	0.103**(0.001)
Park distance	0.067**(0.005)	0.090*(0.012)	−0.014(0.668)
Number of parks	0.037(0.12)	−0.043(0.231)	0.106**(0.01)
Number of overpasses	0.014(0.55)	0.025(0.485)	0.013(0.674)
Number of bus stops	−0.104**(0.000)	−0.066(0.066)	−0.056(0.076)
Streetscape greenery	0.070**(0.003)	0.094**(0.008)	0.034(0.305)

*p < 0.05; **p < 0.01. Bold values represent having relevance.

Total area of Table 2 shows that, amongst the socio-demographic variables, BMI was positively correlated with disease ( r=0.210 ; p=0.000 ) and negatively correlated with age ( r=−0.169 ; p=0.000 ). Of the environmental variables, positive correlations were found with population density ( r=−0.068 ; p=0.004 ), intersection density ( r=0.098 ; p=0.000 ), distance to bus stops ( r=0.100 ; p=0.000 ), distance to park ( r=0.067 ; p=0.005 ) and Streetscape greenery ( r=0.070 ; p=0.00 3). It was negatively correlated with the number of bus stops ( r=−0.104 ; p=0.000 ).

Under different socio-economic indicators, age ( r=−0.164 ; p=0.000 ) was negatively correlated, and the prevalence of chronic illnesses was strongly linked ( r=0.094 ; p=0.008 ) with the BMI of the senior population in the low SES zone. Street connection ( r=−0.072 ; p=0.044 ) and streetscape greenness ( r=0.106 ; p=0.001 ) were adversely and favourably linked, respectively, with the BMI of the older population in regions with low SES. Within the high SES zone, gender ( r=0.082 ; p=0.010 ) was favourably related with the impact of BMI in the older age group, whereas age ( r=−0.155 ; p=0.000 ) was negatively associated and having a chronic disease ( r=0.203 ; p=0.000 ) was positively associated. Population density ( r=−0.103 ; p=0.001 ) was negatively correlated with the BMI of the older adults group in the high SES area; road intersection density ( r=0.073 ; p=0.021 ), distance to bus stops ( r=0.103 ; p=0.001 ) and number of parks ( r=0.106 ; p=0.001 ) all had positive effects on the BMI of the older adults group.

Age and chronic disease effects on BMI in the older adults population were consistent across SES regions. They differed in the variables affecting BMI in the built environment, which is consistent with previous research findings (72).

### 4.3. Results of the linear regression model

Two sets of linear regression models were built: one for the overall older adult group (Table 3) and the other for the comparison of models under different SES (Table 4). Both models were used to explore BMI in the older adult group. The results for the overall older adult group showed that some environmental variables did not correlate with older adult BMI, and the results of the comparison studies within different SES regions were different from previous ones.

**Table 3:** Linear regression model.

Variable	*β*	*t*	*p*-value
(Constant)		19.269	0
Gender	0.036	1.596	0.111
Age	**−0.129**	**−5.575**	**0**
Diseases	**0.201**	**8.75**	**0**
Physical activity	−0.008	−0.352	0.725
Population density	0.022	0.461	0.645
Land use diversity	**0.125**	**3.738**	**0**
Street connectivity	0.037	1.333	0.183
Road intersection density	0.006	0.182	0.856
Distance to park	−0.017	−0.613	0.54
Distance to bus stops	−0.025	−0.777	0.437
Number of bus stops	**−0.178**	**−4.99**	**0**
Number of parks	**0.07**	**2.688**	**0.007**
Number of overpasses	**0.113**	**2.656**	**0.008**
Streetscape green vision	**0.08**	**2.125**	**0.034**

Words in bold are considered as a significant correlation.

**Table 4:** Comparison of linear regression models for different SES regions.

Variable	*β*	*t*	*p*-value	*β*	*t*	*p*-value
	Low SES			High SES		
(Constant)		19.269	0		22.635	0
Gender	−0.012	−0.352	0.725	**0.08**	**2.637**	**0.008**
Age	**−0.119**	**−3.4**	**0.001**	**−0.144**	**−4.731**	**0**
Diseases	**0.21**	**5.888**	**0**	**0.207**	**6.798**	**0**
Physical activity Min/Day	−0.033	−0.961	0.337	0.014	0.457	0.648
Population density	0.017	0.198	0.843	**−0.14**	**−4.23**	**0**
Land use diversity	0.02	0.325	0.745	0.009	0.283	0.777
Street Connectivity	−0.057	−0.738	0.461	**0.097**	**2.936**	**0.003**
Road intersection density	**−0.11**	**−2.023**	**0.043**	0.021	0.511	0.609
Distance to park	**0.206**	**4.194**	**0**	0.046	1.294	0.196
Distance to bus stops	**−0.14**	**−2.676**	**0.008**	0.039	1.191	0.234
Number of bus stops	**−0.161**	**−2.815**	**0.005**	−0.007	−0.083	0.934
Number of parks	0.058	0.626	0.532	0.044	1.018	0.309
Number of overpasses	0.485	0.584	0.559	0.001	0.028	0.977
Streetscape green vision	−0.034	−0.457	0.648	0.011	0.333	0.739

Words in bold are considered as a significant correlation.

Amongst the socio-economic indicator variables in their effect on overall older adult BMI, age ( t=−0.129 ; p=0.000 ) was negatively correlated, a finding that differed from previous research and was positively associated with underlying disease ( t=0.201 ; p=0.000 ). Amongst the effects of built environment variables on overall older adult BMI, land use mix ( t=3.738 ; p=0.000 ), number of parks ( t=2.688 ; p=0.007 ), number of flyovers ( t=2.656 ; p=0.008 ) and streetscape green views ( t=2.125 ; p=0.034 ) were positively associated with the number of bus stops ( t=−4.99 ; p=0.000 ). The *R*^2^ of the base model was 0.093, which indicates that the base model explained 9.3% of the variance.

Table 4 displays the results of linear regression models examining the influence of the built environment on BMI in the older population under various SES situations. Within the low SES region, age was inversely linked with the impact of BMI in the senior population ( t=−119;p=0.001 ), whilst having a chronic condition was favourably associated with BMI in the older adults population ( t=0.210;p=0.000 ); of the built environment variables, road intersection density ( t=−0.110;p=0.043 ), distance to a bus stop ( t=−0.140;p=0.008 ) and the number of bus stops ( t=−0.161;p=0.005 ) were negatively associated with BMI in the older adults group, and distance to a park (t=0.206;p=0.000 ) was favourably related with BMI in the older adults.

Within the high SES region, gender (t=0.080;p=0.008) and chronic illness (t=0.207;p=0.000) were favourably linked with the impact of BMI in the older age group, whilst age (t=−0.144;p=0.000) was adversely associated; population density (t=−0.140;p=0.000) was adversely linked with the impact of BMI in the elder group, but street connectivity was favourably associated with BMI in the older adults group (t=0.097;p=0.003) . The *R*^2^ of the linear regression model for the low SES region was 0.099, which shows that 9.9% of the variation was explained by the basic model; the *R*^2^ of the linear regression model for the high SES region was 0.090, which implies that the base model explained 9.0% of the variance.

## 5. Discussion

In this research, we utilize linear regression to analyze the link between BMI and built environment characteristics in older adults, as well as the influence of built environment variables on BMI in older adult groups residing in various SES locations. Understanding the influence of the built environment on the BMI of older persons will aid in the design of a community setting that is health friendly for older people.

This research discovers that, at the individual level, the variable of age has a negative connection within the general area and in the low SES and high SES sectors, which means that BMI falls with age in the older age group (79). The regression coefficients for the high and low SES regions show that the BMI of the older adults group in the high SES region tends to decline more rapidly with age than that of the older adults group in the low SES region. This finding is consistent with a study conducted by Zang et al. (80). In general and low SES regions, the influence of gender on the BMI of the older adults population is insignificant. In the high SES zone, the correlation is considerably positive, which implies that males are more likely to have a higher BMI and be obese (81). The group of older adults with underlying conditions show a significant positive association with group BMI in older adults within the overall, low SES and high SES regions, which suggests that having underlying conditions can lead to an increase in BMI (82). In previous studies, performing physical exercise leads to a lower BMI. However, the number of hours of physical exercise performed per day is insignificantly correlated with the BMI of the older population within the overall, low SES and high SES regions in our study. Therefore, performing physical exercise per day does not lead to a decrease in BMI, which is possibly due to the fact that the number of hours of physical exercise performed per day is insignificantly correlated with the BMI of the older population within (80); the number of hours of physical exercise performed per day in this study fails to meet the threshold for influencing changes in BMI (83).

In terms of built environment variables, population density has little effect on the BMI of the older population in the region as a whole. Within the low SES region, population density has reached or even exceeded the threshold for promoting travel amongst older adults probably due to low SES region (74). Too high population density is a disincentive for older adults to travel (55, 72). But interestingly, there is a significant negative correlation within high SES areas, because higher population density often means more destinations and services within walking distance, more public transport choices and opportunities for social interaction (84). Moreover, high population density is directly related to more walking trips (85). This may be because these factors can promote active travel among the older adults population in high SES areas, thereby reducing BMI (86, 87).

Land-use admixture in the overall model showed a significant positive correlation with BMI in the older adults. The results are inconsistent with studies in some developed countries, where land use mixing was most strongly associated with obesity in Frank’s study in the United States (65); and in Saelens’ study, people living in communities with high walkability and high land use mixing were associated with lower BMI (88). However, in a study in Hong Kong, land-use admixture was negatively associated with physical exercise in older adults (72); In a study conducted in Shanghai, it was also reported that a negative association between land use mix and physical exercise in older adults (89). Cities with different residential density in different countries may lead to different findings. Therefore, in some cities in China, high land use mixing may reduce the willingness of the older adults to travel, leading to a reduction of physical exercise of the older adults, thus increasing the risk of obesity.

Street connectivity is only positively associated with BMI in older adults in higher SES areas. However, the research demonstrates that street connection has a favorable effect on obesity reduction in densely populated areas (67). The reason may be that better street connectivity can increase the motivation of older adults to walk. However, excessive and complex street connectivity has led to cognitive difficulties for older adults in some studies in China, and this situation reduces their sense of safety and negatively affects their willingness to travel and exercise (90).

Road intersection density is negatively associated with BMI in older adults living in areas with low SES. This result may be due to the fact that high road intersection density improves the travel experience of older adults, which promotes travel and lessens BMI. This finding is coherent with those obtained in some previous studies (80).

Distance to the park is positively correlated with older adults’ BMI in areas with low SES, which may be due to that respondents’ neighborhoods are far from the park and fail to elicit a willingness to travel. Some Chinese studies have shown that poor park accessibility and susceptibility to noise reduce the attractiveness of parks to older adults (91). A positive correlation exists between the number of parks and the BMI of older adults in the overall region. This finding may be due to the fact that local older adults are accustomed to doing activities in familiar neighborhoods and that local parks provide relatively little space for activities that are not conducive to physical activity.

In locations with low SES, BMI is inversely related with proximity to a bus stop amongst older adults persons. In low SES neighborhoods, the frequency of bus stops is inversely related with BMI amongst older persons. The reason may be the poor accessibility of public transport in low SES areas, which increases the probability of travelling on foot and thus reduces BMI (92).

A positive correlation exists between the number of flyovers and the BMI of older individuals in the whole area. This finding may be due to that too many flyovers cause difficulty for older persons to travel, which negatively affects their desire to do so. In contrast to the finding in earlier research, streetscape greenness is positively connected with BMI amongst older persons in the entire area (34, 53). The reason may be due to the lack of street greenery in the respondent’s neighborhood, the inability of green spaces to motivate older adults to exercise and the fact that some of the leisure facilities are situated inside green areas and therefore have no detrimental effect on BMI.

The built environment affects BMI in older adults, and this effect is complex and variable. The results of some studies differ from those of previous works, and more studies of the same type are needed to determine the general applicability of the effect.

## 6. Conclusion

This research highlights the significance of the built environment in a relatively low SES inland city by using Lanzhou, a typical city in Northwest China, as an example. We verify the relationship by assuming that the overall older population and the older population in regions under different SES have a meaningful relationship with the built environment. We use Pearson correlation and linear regression models.

This study has some limitations and restrictions. We only measured built environment attributes around the respondent’s home. However, inhabitants are permitted to go to other locations, such as workplaces and stores. Future research might examine the impact of the built environment on BMI at diverse vacation destinations. Future research might evaluate the combined impacts of physical activity and food environment on obesity given that this study focused only on the community’s built environment. We also did not incorporate all pertinent aspects of the built environment, such as traffic and criminal safety (93, 94). This study was not conducted in a nonlinear manner through a linear research with pre-existing assumptions, lacked the relative importance of environmental characteristics and ignored the effect of some environmental factors on BMI in older age groups.

The data indicate that the age of older persons is negatively connected with BMI and positively correlated with underlying disease throughout the whole area. In terms of environmental characteristics, the number of parks and land use diversity correlate positively with BMI, but the number of bus stops correlates negatively. We detect a higher influence of BMI on the built environment in older age groups in low SES areas than in high SES areas. Attention to environmental planning in low SES areas is more likely to help reduce the risk of obesity in older adults. Our research serves as a guide for urban planners and designers to address the health of older individuals in urban planning.

## Data availability statement

The original contributions presented in the study are included in the article/Supplementary material, further inquiries can be directed to the corresponding author.

## Ethics statement

The studies involving human participants were reviewed and approved by Ethical Review Committee of Guangdong University of Technology. The patients/participants provided their written informed consent to participate in this study.

## Author contributions

PZ: conceptualization. JH: resources. HQ: supervision. HZ: validation. PZ and KC: writing – original draft. KC: writing – review and editing. YY: analysis. All authors contributed to the article and approved the submitted version.

## Funding

This research was funded by National Natural Science Foundation of China (No. 52278014) and Guangzhou Science and Technology Plan Project (No. 2023A04J2012).

## Conflict of interest

The authors declare that the research was conducted in the absence of any commercial or financial relationships that could be construed as a potential conflict of interest.

## Publisher’s note

All claims expressed in this article are solely those of the authors and do not necessarily represent those of their affiliated organizations, or those of the publisher, the editors and the reviewers. Any product that may be evaluated in this article, or claim that may be made by its manufacturer, is not guaranteed or endorsed by the publisher.

## Footnotes

^1^https://en.tongdajiaju.cn/maps.html

## Supplementary material

The Supplementary material for this article can be found online at:

https://www.frontiersin.org/articles/10.3389/fpubh.2023.1207975/full#supplementary-material
