# Supplementary material for: Effect of built environment on BMI of older adults in regions of different socio-economic statuses
Source: Front Public Health. 2023 Jul 7;11:1207975. doi: 10.3389/fpubh.2023.1207975 (PMC10361068; doi:10.3389/fpubh.2023.1207975)
Supplement: Supplementary file 1 [file Table_1.DOCX]

**Supplementary Material**

# Supplementary Material 1:The ICC of the null model

Through the null model modeling of the entire data, the first layer is the community location, and the variance and ICC within the group are tested. The result shows that the ICC is 0.063, so the general linear regression is used for modeling.

Table S1：Intraclass correlation coeffient

| **Estimates of Covariance Parameters^a^** | | | | | | | |
| --- | --- | --- | --- | --- | --- | --- | --- |
| Parameter | | Estimate | Std.Error | Waid Z | Sig. | 95% confidence interval | |
|  |  |  |  |  |  | Low Bound | Upper Bound |
| Residual | | 9.286258 | .313245 | 29.645 | .000 | 8.692165 | 9.920956 |
| Intercept [sbuject= Address] | Variance | 0.618789 | .314184 | 1.970 | .049 | .228746 | 1.673907 |
| a. Dependent Variable：BMI | | | | | | | |

$$\rho=\frac{\sigma_{u0}}{\sigma_{u0}^{2}+\sigma_{e}^{2}}$$

$$ICC=\frac{0.618789}{0.618789+9.2862}=0.0625$$

# Supplementary Material 2:Model

VIF of model and parameters are shown in Table S2.Where VIF is <5, there is no multicollinearity for the independent variables within the model.

Model1 formula :y=24.451+ 1.251*Diseases- 0.052*Age- 0.058*Number_of_Bus_Stops+ 0.202*Number_of_overpasses+ 3.143*Land_use_diversity+ 0.233*Number_of_parks+ 11.262*Streetscape_Green_Vision

Table S2 Model Coefficients

| Coefficients^a^ | | | | | | | | | | |
| --- | --- | --- | --- | --- | --- | --- | --- | --- | --- | --- |
| Model | | Unstandardized Coefficients | | Standardized Coefficients | t | Sig. | 95.0% Confidence Interval for B | | Collinearity Statistics | |
|  |  | B | Std. Error | Beta |  |  | Lower Bound | Upper Bound | Tolerance | VIF |
| 1 | (Constant) | 23.591 | .105 |  | 225.634 | .000 | 23.386 | 23.796 |  |  |
|  | Diseases | 1.305 | .144 | .210 | 9.036 | .000 | 1.022 | 1.588 | 1.000 | 1.000 |
| 2 | (Constant) | 28.132 | .671 |  | 41.919 | .000 | 26.816 | 29.448 |  |  |
|  | Diseases | 1.249 | .143 | .201 | 8.741 | .000 | .968 | 1.529 | .997 | 1.003 |
|  | Age | -.063 | .009 | -.157 | -6.848 | .000 | -.081 | -.045 | .997 | 1.003 |
| 3 | (Constant) | 28.878 | .686 |  | 42.085 | .000 | 27.532 | 30.224 |  |  |
|  | Diseases | 1.322 | .143 | .213 | 9.255 | .000 | 1.042 | 1.603 | .984 | 1.016 |
|  | Age | -.057 | .009 | -.143 | -6.212 | .000 | -.076 | -.039 | .979 | 1.021 |
|  | Number_of_Bus_Stops | -.035 | .008 | -.108 | -4.657 | .000 | -.050 | -.020 | .972 | 1.029 |
| 4 | (Constant) | 28.844 | .685 |  | 42.110 | .000 | 27.500 | 30.187 |  |  |
|  | Diseases | 1.302 | .143 | .210 | 9.121 | .000 | 1.022 | 1.583 | .982 | 1.018 |
|  | Age | -.057 | .009 | -.141 | -6.126 | .000 | -.075 | -.038 | .978 | 1.022 |
|  | Number_of_Bus_Stops | -.048 | .009 | -.147 | -5.442 | .000 | -.065 | -.031 | .714 | 1.401 |
|  | Number_of_overpasses | .133 | .048 | .075 | 2.800 | .005 | .040 | .227 | .726 | 1.378 |
| 5 | (Constant) | 27.380 | .849 |  | 32.239 | .000 | 25.714 | 29.046 |  |  |
|  | Diseases | 1.264 | .143 | .203 | 8.830 | .000 | .983 | 1.545 | .973 | 1.027 |
|  | Age | -.053 | .009 | -.132 | -5.693 | .000 | -.071 | -.035 | .960 | 1.041 |
|  | Number_of_Bus_Stops | -.067 | .011 | -.204 | -6.116 | .000 | -.088 | -.045 | .465 | 2.149 |
|  | Number_of_overpasses | .253 | .063 | .142 | 4.022 | .000 | .130 | .376 | .415 | 2.407 |
|  | Land_use_diversity | 2.274 | .783 | .090 | 2.904 | .004 | .738 | 3.811 | .535 | 1.870 |
| 6 | (Constant) | 26.897 | .882 |  | 30.491 | .000 | 25.167 | 28.627 |  |  |
|  | Diseases | 1.257 | .143 | .202 | 8.787 | .000 | .976 | 1.537 | .973 | 1.028 |
|  | Age | -.053 | .009 | -.132 | -5.705 | .000 | -.071 | -.035 | .960 | 1.041 |
|  | Number_of_Bus_Stops | -.067 | .011 | -.205 | -6.175 | .000 | -.089 | -.046 | .465 | 2.150 |
|  | Number_of_overpasses | .286 | .065 | .160 | 4.402 | .000 | .158 | .413 | .389 | 2.573 |
|  | Land_use_diversity | 2.558 | .795 | .101 | 3.216 | .001 | .998 | 4.118 | .518 | 1.931 |
|  | Number_of_parks | .159 | .079 | .048 | 2.004 | .045 | .003 | .315 | .891 | 1.122 |
| 7 | (Constant) | 24.451 | 1.450 |  | 16.866 | .000 | 21.607 | 27.294 |  |  |
|  | Diseases | 1.251 | .143 | .201 | 8.750 | .000 | .970 | 1.531 | .972 | 1.028 |
|  | Age | -.052 | .009 | -.129 | -5.575 | .000 | -.070 | -.034 | .957 | 1.045 |
|  | Number_of_Bus_Stops | -.058 | .012 | -.178 | -4.990 | .000 | -.081 | -.035 | .404 | 2.476 |
|  | Number_of_overpasses | .202 | .076 | .113 | 2.656 | .008 | .053 | .351 | .283 | 3.529 |
|  | Land_use_diversity | 3.143 | .841 | .125 | 3.738 | .000 | 1.494 | 4.792 | .462 | 2.162 |
|  | Number_of_parks | .233 | .087 | .070 | 2.688 | .007 | .063 | .403 | .749 | 1.335 |
|  | Streetscape_Green_Vision | 11.262 | 5.299 | .080 | 2.125 | .034 | .869 | 21.655 | .362 | 2.766 |
| a. Dependent Variable: BMI | | | | | | | | | | |

# Supplementary Material 3:Wilcoxon analysis of different SES

Table S3 examines the difference between high SES and low SES regions, and proves that there are significant differences between the two from a statistical point of view, so it is divided into two models of high and low economic regions for discussion.

| Table S3 Paired sample Wilcoxon analysis results | | | | | |
| --- | --- | --- | --- | --- | --- |
| name | paired median M (P25，P75) | | Median M difference (pair 1-pair 2) | Statistics z-value | *p* |
|  |  | |  |  |  |
|  | Paired 1 | Paired 2 |  |  |  |
| High SES *paired*  Low SES | 23.051(21.6,24.5) | 24.446(22.5,26.7) | -1.395 | 24.170 | 0.000** |
| * *p*<0.05 ** *p*<0.01 | | | | | |

# Supplementary Material 4:Wilcoxon analysis of different SES

TableS4 uses ten-fold cross-validation to test the evaluation indicators of the linear model and the nonlinear model. MAE and RMSE prove that there is a significant difference between the two values ​​​​from a statistical point of view. The smaller the two evaluation indicators, the better. Therefore, we chose a linear regression model.

Table S4 Model comparison between linear regression and GBDT

| Model | Median(P25,P75) | Z | p |
| --- | --- | --- | --- |
| Linear regression model MAE | 2.350(2.3,2.4) | 2.803 | 0.005** |
| GBDT regression model MAE | 2.368(2.4,2.4) |  |  |
| Linear regression RMSE | 2.984(3.0,3.0) | 2.803 | 0.005** |
| GBDT regression RMSE | 3.024(3.0,3.0) |  |  |
